# Knee Pain, Joint Loading, and Structural Abnormalities on MRI in 13-Year-Old Children in a Population-Based Birth Cohort

**DOI:** 10.1177/03635465241274792

**Published:** 2024-09-25

**Authors:** Guido J. van Leeuwen, Laura A.M. Kemmeren, Tom M. Piscaer, Edwin H.G. Oei, Patrick J.E. Bindels, Sita M.A. Bierma-Zeinstra, Marienke van Middelkoop

**Affiliations:** † Department of General Practice, Erasmus MC University Medical Center Rotterdam, Rotterdam, The Netherlands; ‡ Department of Orthopedics and Sports Medicine, Erasmus MC University Medical Center Rotterdam, Rotterdam, The Netherlands; § Department of Radiology and Nuclear Medicine, Erasmus MC University Medical Center Rotterdam, Rotterdam, The Netherlands; Investigation performed at Erasmus MC University Medical Center Rotterdam, Rotterdam, The Netherlands

**Keywords:** epidemiology, knee (general), imaging (magnetic resonance), anatomy, statistics

## Abstract

**Background:**

Knee pain is a common problem in children and adolescents, and it often has a chronic character.

**Purpose:**

To examine the prevalence of knee pain in 13-year-old children and assess associations of knee pain with physical factors and the presence of structural abnormalities on magnetic resonance imaging (MRI).

**Study Design:**

Cross-sectional study; Level of evidence, 3.

**Methods:**

Data from the Generation R Study, a population-based birth cohort, were used. Prevalence and characteristics of knee pain were assessed, using a pain mannequin, in children 13 years of age (N = 1849). Patient characteristics and data on physical activity were extracted from questionnaires. The body mass index standard deviation score and waist-hip ratio were calculated from objectively measured weight and height. Structural abnormalities were assessed by MRI. The differences between children with and without knee pain were also analyzed.

**Results:**

A prevalence of 8.0% was found for knee pain in children, of which 92.3% persisted for >3 months (ie, chronic); 37.5% of the children experienced pain daily, and the pain was almost always located on the anterior side of the knee (98.6%). Higher body mass index standard deviation scores were seen in children with knee pain than in the children without knee pain. No differences in physical activity were seen between children with and without knee pain. Moreover, in children with knee pain compared with children without knee pain, characteristics of Osgood-Schlatter disease (6.8% vs 1.9%) and bipartite patella type 3 (4.7% vs 0.3%) were more often seen on MRI.

**Conclusion:**

This study shows that knee pain is a relatively frequent problem in children. It is almost always located on the anterior aspect, has a chronic character, and is often experienced daily. However, the possible implication of structural abnormalities on MRI in children with knee pain and the possible relationship with the development of future knee complaints are still unclear.

Studies have shown that musculoskeletal pain is already evaluated in children and adolescents and often has the tendency to persist for >2 years and to be experienced daily.^17,34,35^ Despite knee pain being viewed as one of the most common types of musculoskeletal pain, reported prevalence rates in children and adolescents vary greatly, ranging from 7% to 32%.^11,26,35^

It has been suggested that skeletal growth and maturation play an important role in the development of knee pain and other symptoms.^12^ In addition to growth and maturity, various joint loading factors, such as obesity, physical activity, and exercise, are suggested to be associated with the development and presence of musculoskeletal problems in children and adolescents.^14,20,28^ Specifically, more ankle, foot, and knee problems occur in children with overweight or obesity.^20^ However, most studies done so far in this field are of a cross-sectional nature and have a moderate to low quality of evidence.^14,28^ Various nontraumatic knee complaints, such as Osgood-Schlatter disease (OSD) and patellofemoral pain, are known to arise during puberty and have a higher prevalence in physically active children and adolescents.^9,16,36^ In addition, a Danish primary school cohort study and a systematic review have suggested a relationship between a high level of physical activity and exercise and the development of low back and spinal pain in children and adolescents.^6,14^ However, evidence remains conflicting, because, for example, a cohort study in 14-year-old children did not find a relationship between moderate or vigorous physical activity and musculoskeletal pain.^31^ For both loading factors, that is, obesity and physical activity, there seems to be an absence of high-quality prospective studies on their association with knee pain, and evidence for their proposed potential as risk factors in the development in knee pain is lacking.

Several structural changes and features have been proposed as causative factors of knee pain in children and adolescents. For example, OSD is characterized by structural changes such as thickening of the patellar tendon, enlargement of the tibial tuberosity, and a secondary ossification center on radiography.^9^ In adults, joint loading factors such as physical activity and obesity are known to affect knee structure and alignment.^2,5,10,33^ It is, however, unclear if these joint loading factors also lead to structural changes in the knee in children and adolescents that could result in knee problems at an older age. Thus, gaining more insight into the relationship between structural changes and abnormalities on magnetic resonance imaging (MRI) in children and adolescents with knee pain could provide more understanding in the development and pathology of knee pain in children and adolescents.

Therefore, the aim of this study was to describe (1) the prevalence and characteristics of knee pain in 13-year-old children; (2) the association between joint loading factors such as physical activity, sports participation, and weight-related factors and the presence of knee pain; and (3) the presence of structural abnormalities on MRI.

## Methods

### Study Population

This study was performed within the Generation R Study, a population-based prospective cohort study focusing on growth, development, and health from fetal life until young adulthood. Eligible participants were the children of women with an expected delivery date between April 2002 and January 2006, living in Rotterdam, the Netherlands. At the start of the study, 9749 children of the pregnant mothers were included in the cohort. At regular time points during infancy, childhood, and puberty, questionnaires were sent out and physical examination was performed. Detailed information on the Generation R Study cohort has been described elsewhere.^18^ The Medical Ethics Committee of the Erasmus Medical Center, Rotterdam approved the study, and written informed consent was obtained from the parents of all participants.

### Procedures

For the present study, data from the follow-up phases at the ages of 9 and 13 years were used. In total, 6841 children still participated in the study at the age of 13 years, 70% of the original cohort. Data were derived from physical examinations at the research center and self- and parent-reported questionnaires. Questions on pain were asked with a self-reported questionnaire during a second visit at the research center, which was arranged for the purpose of obtaining MRI scans. During the regular first visit at 13 years, a random subset of children received an additional invitation to participate in the knee MRI study. Children who did not participate in the regular first visit did not receive an invitation, and those who declined to participate in the knee MRI study were excluded from the study population. This resulted in a final study population of 1849 children with data on the presence of pain and pain locations and who underwent knee MRI (Appendix Figure A1, available in the online version of this article). Additional questions on pain characteristics were added to the pain questionnaire at a later stage, after delayed ethics approval procedures, and are therefore only available for 325 children.

### Measurements: Questionnaires

#### Demographics

Information on the child's sex was obtained from the midwife and hospital records at birth, and age was registered during the visit.

#### Knee Pain

To assess the presence of pain, children were asked whether they had pain in the past 6 weeks. If so, the location was checked on a pain mannequin with 61 possible locations, at both the front and back sides of the body. In this study, knee pain was defined as pain in the front or back of the left or right knee. No knee pain was defined as children without pain or children with pain in any other pain locations not including the knee (Appendix Figure A2, available online). Additional questions on pain characteristics (available for 325 participants) included the frequency of occurrence (constant, daily, multiple days per week, weekly, monthly, or less than monthly), pain duration (number of weeks or months), onset of pain (sudden or gradual), relationship to sports (yes or no), and pain intensity (numeric rating scale 0-10). Pain was classified as chronic if the reported pain duration was >3 months.

#### Physical Activity

Questionnaire data were used to evaluate physical activity. Physical activity behavior was evaluated by assessing sports participation (yes or no), active transport to and from school, and the amount of physical activity (amount of days per week with at least 1 hour of physical activity). The cutoff for a child being physically active was defined as at least 1 hour per day for at least 4 days per week (ie, more than half of the week). Active transport to and from school was defined as having at least 1 trip of walking or cycling to school per week.

For the history of physical activity and sports participation, the parent-reported questionnaire at the age of 9 years was used. In that questionnaire, parents were asked if their children participated in sports and how many hours per week their children spent doing sports. Being physically activity at the age of 9 was defined as 2 to 4 hours of physical activity (sports, physical education, and active transport) per week.

### Measurements: Physical Examination

#### Anthropometry

Child height was measured in the standing position using a Harpenden stadiometer (Holtain Limited), and weight was measured without heavy clothing and shoes using a mechanical personal scale (SECA). The waist and hip circumferences were measured using a measuring tape (SECA). Height and weight were used to calculate the body mass index (BMI). BMI standard deviation (BMI SD) scores account for child age and sex and were calculated based on the Dutch reference growth curves and cutoffs by Cole and Lobstein,^3^ where a BMI SD score of 0 correlates with a child being in the 50th percentile.^7^ Weight status was categorized according to the cutoffs by Cole and Lobstein^3^ and dichotomized to underweight or normal weight versus overweight or obese. The cutoff for overweight was set at the 90th percentile, which correlates with a BMI SD score of roughly 1.3.^3,7^ Waist and hip circumferences were used to calculate the waist-hip ratio (WHR). WHR was categorized according to the cutoffs by Fredriks et al^8^ and dichotomized to underweight or normal weight versus overweight or obese.

### Measurements: MRI

All MRI scans were performed using a dedicated 3-T MRI scanner (Discovery MR750; GE Healthcare), and children were scanned according to standard imaging and positioning protocols for the Generation R Study.^18^ The participants’ knees were scanned using a water excitation GRASS (gradient recalled acquisition in the steady state) 3-dimensional (3D) sequence and a LAVA (liver acquisition with volume acceleration) Flex sequence, providing in-phase and out-phase imaging.^24^ The slice thickness was 0.7 mm for the GRASS 3D sequence and 1.0 mm for the LAVA Flex sequence. For both sequences, the in-plan (reconstructed) voxel size was 0.7 × 0.7 mm^2^.

The following scoring list was developed based on the literature and expert opinion: central or complete physis closure, tibia crack ossification, tibia epiphysis closure, fibula styloid ossification.^29^ Presence of bone edema, bipartite patella (graded as type 1, inferior; type 2, lateral; or type 3, superolateral),^19^ and signs of OSD (graded as the radiological presence of edema of the tibial tuberosity, distal patellar tendon, or adjacent fat and/or the presence of an ossicle of the tibial tuberosity).^13,23^ Furthermore, characteristics of Sinding-Larsen-Johansson (SLJ) syndrome were noted (graded as edema of the proximal patellar tendon, inferior pole of the patella, or adjacent fat or the presence of an ossicle of the inferior pole of the patella).^21^

MRI scans were assessed by an orthopaedic resident and PhD student (L.A.M.K.), after training by a senior musculoskeletal radiologist (E.H.G.O.) and a senior orthopaedic surgeon specialized in knee surgery (T.M.P.). All abnormalities that were found were reevaluated by T.M.P. When in doubt, E.H.G.O. was consulted and agreement was reached between the 3 authors. All were blinded to all participant characteristics, such as sex, height, and weight, during the assessment of the MRI scans. A random sample of 50 MRI scans was taken and evaluated by both L.A.M.K. and T.M.P. to assess interobserver reliability.

### Statistical Analysis

Child characteristics and prevalence of pain were described using descriptive statistics. To analyze the differences between children with knee pain and children without knee pain (ie, no knee pain), the chi-square or Fisher exact test was used for categorical variables, structural features, and analysis of variance; for continuous variables, the Mann-Whitney *U* (2 groups) and Kruskal-Wallis (>2 groups) tests were used. Post hoc analyses were performed to assess which of the 2 subgroups significantly differed from each other. All analyses on the differences between children with knee pain and children without knee pain and structural features on MRI were only performed if the total number of children per feature was >10. To estimate the interobserver reliability of the MRI assessment, the prevalence-adjusted bias-adjusted kappa (PABAK) was calculated. SPSS software (Version 28.0; IBM Corp) was used for all analyses, and the level of statistical significance was set at *P* < .05.

## Results

### Participant Characteristics

More than half (51.8%) of the 1849 included children were girls (Table 1). The children had a median age of 13.8 years (IQR, 13.6-14.6 years), with a BMI SD score of 0.43 (SD, 1.19), and almost one-third (28.0%) had an overweight or obese WHR. Compared with the children who did not participate in the MRI study, participants in this study were significantly less often boys, had an older age, and had a lower BMI (Appendix Table A1, available online).

**Table 1:** Participant Characteristics^*a*^

	Total (N = 1849)	Knee Pain (n = 148)	No Knee Pain (n = 1701)	*P* Value
Demographics				
Female sex	958 (51.8)	88 (59.5)	870 (51.1)	.052
Age, y	13.84 [13.61-14.56]	13.83 [13.59-14.35]	13.84 [13.61-14.57]	.319
Physical factors				
BMI SD score	0.43 {1.19}	0.61 {1.17}^*b*^	0.41 {1.20}^*b*^	**.050**
Overweight	293 (15.9)	28 (18.9)	265 (15.6)	.289
Height score	0.002 {1.02}	0.04 {0.97}	–0.001 {1.03}	.649
Waist-hip ratio overweight or obese	506 (28.0)	42 (28.8)	464 (27.9)	.820
Physical activity behaviors				
Physical activity, minimum 1 h/day ≥4 days/wk	875 (66.1)	74 (66.7)	801 (66.0)	.893
Sports participation	1298 (84.8)	108 (86.4)	1190 (84.6)	.599
Active transport, ≥1 trip/wk	1130 (88.1)	89 (83.2)	1041 (88.6)	.097
History of physical activity behaviors				
Physical activity at 9 y	918 (69.3)	76 (71.7)	842 (69.1)	.574
Sports participation at 9 y	1304 (89.1)	104 (88.9)	1200 (89.2)	.930

a Values are presented as n (%) for categorical factors or median [IQR] or mean {SD} for continuous factors. Bold values represent statistically significant *P* values (*P* < .05). This table is based on nonimputed data. Missing values were 0 for sex; 0 for age; 2 (0.1%) for body mass index (BMI), overweight, and height; 39 (2.1%) for waist-hip ratio; 525 (28.4%) for physical activity; 318 (17.2%) for sports participation; 567 (30.7%) for active transport; 524 (28.3%) for physical activity at 9 years; and 386 (20.9%) for sports at 9 years. BMI SD, body mass index standard deviation.

b Subgroups that significantly differ from each other, based on post hoc analyses.

### Reporting of Knee Pain

In total 148 children (8.0%) reported knee pain in the previous 6 weeks.

From the children who experienced knee pain (8.0%), almost all reported pain at the ventral side of the knee (98.6%), almost half of the children reported bilateral pain (45.9%), 37.5% experienced knee pain daily, and 92.3% of the children with knee pain experienced chronic pain (>3 months). Moreover, almost three-quarters of the children reporting knee pain indicated that the pain was related to sports (73.4%). Of the children with knee pain, 28.4% also experienced pain at other musculoskeletal sites, most often in the ankle and foot (14.2%) or the back (8.8%).

Only 6.8% of the children experienced pain at nonmusculoskeletal sites, mainly in the head (4.1%) and stomach (3.4%). There were no differences in pain characteristics between boys and girls (Table 2).

**Table 2:** Pain Characteristics in Knee Pain Group^*a*^

	Total (n = 148)	Boys (n = 60)	Girls (n = 88)	*P* Value
Location of knee pain				
Ventral	146 (98.6)	59 (98.3)	87 (98.9)	.784
Dorsal	19 (12.8)	8 (13.3)	11 (12.5)	.882
Bilateral	68 (45.9)	29 (48.3)	39 (44.3)	.630
Frequency of occurrence				
<1/mo	7 (10.9)	3 (12.5)	4 (10.0)	.315
≥1/mo, <1/wk	3 (4.7)	1 (4.2)	2 (5.0)	
≥1/wk	30 (46.9)	8 (33.3)	22 (55.0)	
Every day	24 (37.5)	12 (50.0)	12 (30.0)	
Pain intensity, NRS score	5.70 {1.58}	5.92 {1.25}	5.58 {1.75}	.407
Pain duration, >3 mo	48 (92.3)	15 (93.8)	33 (91.7)	.795
Onset				
Sudden	35 (56.5)	12 (52.2)	23 (59.0)	.602
Gradual	27 (43.5)	11 (47.8)	16 (41.0)	
Related to sports	47 (73.4)	16 (66.7)	31 (77.5)	.342
Pain at other sites				
Other musculoskeletal	42 (28.4)	12 (20.0)	30 (34.1)	.062
Neck	00 (0.0)	00 (0.0)	00 (0.0)	.566
Shoulder	3 (2.0)	2 (3.3)	1 (1.1)	.242
Back	13 (8.8)	3 (5.0)	10 (11.4)	.402
Hip	6 (4.1)	1 (1.7)	5 (5.7)	.228
Ankle/foot	21 (14.2)	6 (10.0)	15 (17.0)	.202
Other	10 (6.8)	2 (3.3)	8 (9.1)	
Head	6 (4.1)	2 (3.3)	4 (4.5)	>.999
Stomach	5 (3.4)	2 (3.3)	3 (3.4)	>.999
Chest	2 (1.4)	00 (0.0)	2 (2.3)	.515

a Values are presented as n (%) for categorical factors or mean {SD} for continuous factors. This table is based on nonimputed data. Missing values were 0 for location of pain, 84 (56.8%) for frequency of occurrence, 84 (56.8%) for pain intensity, 96 (64.9%) for pain duration, 86 (58.1%) for pain onset, 84 (56.8%) for pain related to sports, and 0 for pain at other sites. NRS, numeric rating scale.

### Differences Between Children With and Without Knee Pain

In Table 1, differences in demographics and physical and psychosocial factors between the groups of children with and without knee pain are presented. Children with knee pain were more often girls than children without knee pain (59.5% vs 51.5%), and also higher BMI SD scores were seen in children with knee pain compared with children without knee pain (0.61 [SD, 1.17] vs 0.41 [SD, 1.20]). There were no differences in physical activity behaviors at 13 or 9 years of age between children with and without knee pain.

### Differences in MRI Abnormalities Between Children With and Without Knee Pain

The PABAK was 0.84 for finding any abnormality, indicating good interobserver reliability. There were no differences in central physis closure, complete physis closure, or the presence of edema between children with and without knee pain (Table 3). In this study, 34 children (1.8%) had tibia crack ossification, which was more frequently present in children with knee pain than children without knee pain (4.7% vs 1.6%). Moreover, characteristics of OSD, both edema and an ossicle of the tibial tuberosity, were found in 43 children (2.3%) and were seen more often in children with knee pain compared with children without knee pain (6.8% vs 1.9%). Furthermore, 15 children (0.8%) with knee pain had characteristics of SLJ on MRI and, in contrast with the children without knee pain, had more edema associated with SLJ (2.7% vs 0.5%). Lastly, in 8 children (5.4%) with knee pain bipartite patella was found and compared with children without knee pain more bipartite patella type 3 was seen (4.7% vs 0.3%).

**Table 3:** Descriptive Statistics on MRI Abnormalities Seen in the Knees of 13-Year-Old Children^*a*^

	Total (N = 1849)	Knee Pain (n = 148)	No Knee Pain (n = 1701)	*P* Value
Central physis closure				
Central ≥1 location	429 (23.2)	38 (25.7)	391 (23.0)	.457
Tibia	425 (23.0)	38 (25.7)	387 (22.8)	.417
Femur	195 (10.5)	17 (11.5)	178 (10.5)	.698
Fibula	227 (12.3)	21 (14.2)	206 (12.1)	.460
Complete physis closure				
Complete ≥1 location	1754 (94.9)	141 (95.3)	1613 (94.8)	.815
Tibia	151 (8.2)	10 (6.8)	141 (8.3)	.514
Femur	40 (2.2)	3 (2.0)	37 (2.2)	.905
Fibula	913 (49.4)	83 (56.1)	830 (48.8)	.089
Patella	1754 (94.9)	141 (95.3)	1613 (94.8)	.815
Tibia crack ossification	34 (1.8)	7 (4.7)^*b*^	27 (1.6)^*b*^	**.006**
Tibia epiphysis closure	1439 (77.8)	112 (75.7)	1327 (78.0)	.512
Fibula styloid ossification	913 (49.4)	83 (56.1)	830 (48.8)	.089
Edema				
≥1 location, yes	133 (7.2)	16 (10.8)	117 (6.9)	.076
Tibia plateau	26 (1.4)	1 (0.7)	25 (1.5)	.431
Tibia	12 (0.6)	2 (1.4)	10 (0.6)	.267
Femoral condyles	84 (4.5)	10 (6.8)	74 (4.4)	.178
Femur	1 (0.1)	00 (0.0)	1 (0.1)	
Fibula	7 (0.4)	1 (0.7)	6 (0.4)	
Patella	23 (1.2)	4 (2.7)	19 (1.1)	.095
Characteristics of Osgood-Schlatter disease				
≥1 characterization	43 (2.3)	10 (6.8)^*b*^	33 (1.9)^*b*^	**<.001**
Edema ≥1 location	40 (2.2)	10 (6.8)^*b*^	30 (1.8)^*b*^	**<.001**
Ossicle tibial tuberosity	18 (1.0)	6 (4.1)^*b*^	12 (0.7)^*b*^	**<.001**
Characteristics of Sinding-Larsen-Johansson syndrome				
≥1 characterization	15 (0.8)	4 (2.7)^*b*^	11 (0.6)^*b*^	**.007**
Edema ≥1 location	13 (0.7)	4 (2.7)^*b*^	9 (0.5)^*b*^	**.002**
Ossicle inferior pole patella	5 (0.3)	1 (0.7)	4 (0.2)	
Fragmentation inferior pole patella	00 (0.0)	00 (0.0)	00 (0.0)	
Bipartite patella				
Type 1/2/3	18 (1.0)	8 (5.4)^*b*^	10 (0.6)^*b*^	**<.001**
Type 1	2 (0.1)	00 (0.0)	2 (0.1)	
Type 2	5 (0.3)	1 (0.7)	4 (0.2)	
Type 3	12 (0.6)	7 (4.7)^*b*^	5 (0.3)^*b*^	**<.001**

a Values are presented as n (%). Bold values represent statistically significant *P* values (*P* < .05). This table is based on nonimputed data. MRI, magnetic resonance imaging.

b Subgroups that significantly differ from each other, based on post hoc analyses. Analyses were only performed if the total number of children per feature was >10.

## Discussion

In this study, a prevalence of 8.0% was found for knee pain in children aged 13 years, of which >90% was chronic (ie, lasted >3 months) and almost half of the children experienced daily pain. Knee pain was more frequently reported by children with a higher BMI compared with children without knee pain. Characteristics of OSD and bipartite patella type 3 were seen more often on MRI in children with knee pain compared with children without knee pain. Lastly, in the children with knee pain, tibia crack ossification and SLJ edema were seen more often than in children without knee pain.

The 8% prevalence of knee pain found in this study is lower than previously reported prevalence rates of 25% to 32.3% in Danish children and adolescents.^26,35^ The lower prevalence of knee pain in children found in this study could be because of the difference in study populations (ie, older high school students and possible responder bias given the open population surveys) between other studies and this study.^11,26,35^ Another explanation is the definition of pain and its strict criteria in the applied questionnaire, namely pain on more than half of the days in the last 6 weeks. Because children with more short-term or intermittent complaints do not meet this criterion, only the more significant or chronic cases of knee pain are counted as knee pain cases in this study. This is also supported by the fact that almost all children with knee pain in this study had chronic, almost daily pain and a relatively high pain intensity, which is in concordance with literature on musculoskeletal and knee pain in children and adolescents.^30,34,35^ Because the results of this study show that knee pain is a common problem in 13-year-old children, and is almost always a chronic and daily complaint, it is important to gain more insight into the precise characteristics and course of knee pain and the characteristics of children with knee pain and their (primary) care seeking behavior. This could lead to a better understanding of these chronic complaints and possibly lead to better signaling of children at risk, which may result in more specific and personalized treatment and minimize the extent of the complaints.

In concordance with the literature, the children with knee pain had a higher BMI SD score than children with no pain.^28^ It is known that being overweight or obese affects joint health, leading to more knee, ankle, and foot problems than in normal weight adults.^2,5,20^ Moreover, research has shown that structural spinal abnormalities normally seen in adults with overweight and obesity status are already seen on MRI in 9-year-old children with a higher weight status.^39^ However, for the knee there is an absence of insight into the presence of structural knee features associated with weight status in children and adolescents, possibly because of a lack of research and, more specifically in this study, because children and adolescents might be too young for specific knee abnormalities related to knee pain in adults to develop and be visible on imaging. In young and middle-aged adults with persistent patellofemoral pain, for example, features of patellofemoral osteoarthritis can already be seen on radiographs and MRI scans, especially in adults with a higher BMI.^4^ Longitudinal research into the presence of structural knee features and possible association with weight status in children with knee pain could provide more insight into the early development of knee problems at a later age.

Many musculoskeletal problems seem to arise due to physical changes and skeletal maturation during puberty.^15,40^ Because the children in this study are a mean age of 13 years and most likely in the middle of puberty, a possible explanation for the reported pain is the physical changes during puberty and pain associated with growing.^27,40^ These pains are associated with physis closure, which increases with age and commences at an earlier age in girls.^37,40^ In this study, children with knee pain in general had more central and complete physis closure, although not significantly, than children without knee pain. This might be because of the differences in sex between the pain groups, because children in the knee pain group consisted of more girls than the group without knee pain. It is known that in girls entering puberty and the accompanying growth spurts, physis closures commence at an earlier age than in boys.^29,37^ Furthermore, the slightly higher percentage of physis closure in the knee pain group also seems to be in agreement with literature showing an increased prevalence of musculoskeletal pain with age and a higher prevalence of musculoskeletal pain among females.^17,36^ Despite the presumed relationship between growth and knee pain in children and adolescents, this association has never been adequately studied. Longitudinal studies that investigate the age distribution of physis closures and the presence of knee pain and the possible association with joint loading factors could provide more insight into the possible association of physis closures and the development of knee pain. This will lead to an increased understanding in the development of knee pain in children and adolescents.

More characteristics of OSD and bipartite patella on MRI were seen in children with knee pain, which is surprising, as bipartite patella is generally thought of as asymptomatic.^19,22^ OSD is a structural feature associated with being physically active, while symptomatic cases of bipartite patella have also been described in children who are highly physically active.^9,25^ Nevertheless, in this study, children with bipartite patella and knee pain were not more physically active than children with bipartite patella without knee pain (data not presented). Furthermore, OSD also occurs more often in physically active individuals and is defined specifically by knee pain, especially during joint loading activities of the knee.^9^ This was reflected in our study results, as the percentage of physically active children with knee pain and OSD characteristics was higher compared with the percentage in children without knee pain, while sports participation was only slightly higher (data not presented). Moreover, no differences were observed in physical activity between children with and without knee pain. This might be because of the relatively high amount of physical activity and sports participation in the study population, making it difficult to find an association given the rather low variability. In conclusion, apart from OSD there is still no clear answer on the possible association between physical activity, knee pain, and structural features. It is important to completely understand this association for early detection and possible interventions in children and adolescents with knee pain. Studies that look into the relationship between intensity, physical load, and the presence of structural changes of the knee could further elucidate the possible association between physical activity, structural features, and the development of knee pain.

### Strengths and Limitations

This study has several strengths, namely the prospective population-based design and the availability of information on several factors studied within the same study sample with a large sample size. Because of the design of the study, data from questionnaires at different time points could be included. This study provides insight into multiple structural features of the knee that can be found at this age and their relationship with knee pain and other physical factors, which to our knowledge has never been done before. A limitation, as known in the general follow-up of the Generation R Study,^18^ is that selection bias toward a more healthy, Western, and higher socioeconomic status population might have occurred, and thus there could be an underestimation of pain because those of lower socioeconomic status could be more likely to report pain.^32^ As discussed, this study used strict criteria for the definition of pain in the questionnaire, which may have led to the inclusion of only the more significant or chronic cases. This nonetheless seems acceptable from a clinical perspective, as these are most likely the patients who would seek care. Another possible limitation might be that girls and boys are included in the same analysis, because girls and boys have differing timelines for attaining skeletal maturity. However, presenting the data for the pain groups separate for girls and boys would yield very low numbers, making interpretation difficult. Moreover, in this study the MRI protocol did not include fast spin echo T1- and T2-weighted sequences, which are commonly used in clinical protocols. Although the applied water excitation GRASS sequence was found to be highly fluid sensitive, this could have led to over- or underrepresentation of certain conditions, for example, bone edema or soft tissue edema. Lastly, study participants reported the location of knee pain on either the front or back of the mannequin (Appendix Figure A1, available online). Unfortunately, it did not contain a knee pain location chart,^1,38^ which could have provided more information on the precise location of pain in the knee in relation to specific features.

## Conclusion

This study is the first study to look into the presence of features on knee MRI in 13-year-old children with knee pain and shows that knee pain is a relatively frequent problem in this study population, and pain is almost always located on the ventral side and often has a chronic character with problems experienced daily. Knee pain is more often seen in children with a higher BMI, with more physis closure, and with characteristics of OSD, edema associated with SLJ, bipartite patella, and tibia crack ossification on MRI. Furthermore, there were no differences in physical activity behaviors between the children with and without knee pain. At this moment, the possible implication of structural features on MRI in children with knee pain and the possible relationship with the development of future knee complaints is still unclear. Therefore, there currently is no reason to refrain from symptomatic nonoperative care and start routinely performing imaging in young patients with (chronic) nontraumatic knee complaints, because there still is no added benefit in the diagnosis or treatment of these complaints. Longitudinal studies into age distribution and the presence of these knee features in children and adolescents and their relationship with knee pain could provide even more insight into the origins and development of knee problems in children and adolescents.

## Supplemental Material

sj-pdf-1-ajs-10.1177_03635465241274792 – Supplemental material for Knee Pain, Joint Loading, and Structural Abnormalities on MRI in 13-Year-Old Children in a Population-Based Birth Cohort Supplemental material, sj-pdf-1-ajs-10.1177_03635465241274792 for Knee Pain, Joint Loading, and Structural Abnormalities on MRI in 13-Year-Old Children in a Population-Based Birth Cohort by Guido J. van Leeuwen, Laura A.M. Kemmeren, Tom M. Piscaer, Edwin H.G. Oei, Patrick J.E. Bindels, Sita M.A. Bierma-Zeinstra and Marienke van Middelkoop in The American Journal of Sports Medicine

## References

[1] Boudreau SA Badsberg S Christensen SW Egsgaard LL . Digital pain drawings: assessing touch-screen technology and 3D body schemas. Clin J Pain. 2016;32(2):139-145. 25756558 10.1097/AJP.0000000000000230

[2] Brouwer GM van Tol AW Bergink AP , et al. Association between valgus and varus alignment and the development and progression of radiographic osteoarthritis of the knee. Arthritis Rheum. 2007;56(4):1204-1211. 17393449 10.1002/art.22515

[3] Cole TJ Lobstein T . Extended international (IOTF) body mass index cut-offs for thinness, overweight and obesity. Pediatr Obes. 2012;7(4):284-294. 22715120 10.1111/j.2047-6310.2012.00064.x

[4] Collins NJ Oei EHG de Kanter JL Vicenzino B Crossley KM . Prevalence of radiographic and magnetic resonance imaging features of patellofemoral osteoarthritis in young and middle-aged adults with persistent patellofemoral pain. Arthritis Care Res. 2019;71(8):1068-1073. 10.1002/acr.23726 30133185

[5] Felson DT Goggins J Niu J Zhang Y Hunter DJ . The effect of body weight on progression of knee osteoarthritis is dependent on alignment. Arthritis Rheum. 2004;50(12):3904-3909. 15593215 10.1002/art.20726

[6] Franz C Møller NC Korsholm L Jespersen E Hebert JJ Wedderkopp N . Physical activity is prospectively associated with spinal pain in children (CHAMPS Study-DK). Sci Rep. 2017;7(1):11598. 28912463 10.1038/s41598-017-11762-4 PMC5599496

[7] Fredriks AM van Buuren S Burgmeijer RJ , et al. Continuing positive secular growth change in The Netherlands 1955-1997. Pediatr Res. 2000;47(3):316-323. 10709729 10.1203/00006450-200003000-00006

[8] Fredriks AM van Buuren S Fekkes M Verloove-Vanhorick SP Wit JM . Are age references for waist circumference, hip circumference and waist-hip ratio in Dutch children useful in clinical practice? Eur J Pediatr. 2005;164(4):216-222. 15662504 10.1007/s00431-004-1586-7

[9] Gholve PA Scher DM Khakharia S Widmann RF Green DW . Osgood Schlatter syndrome. Curr Opinion Pediatr. 2007;19(1):44-50. 17224661 10.1097/MOP.0b013e328013dbea

[10] Hanna F Teichtahl AJ Bell R , et al. The cross-sectional relationship between fortnightly exercise and knee cartilage properties in healthy adult women in midlife. Menopause. 2007;14(5):830-834. 17413649 10.1097/gme.0b013e31802f316b

[11] Hartvigsen J Davidsen M Hestbaek L Sogaard K Roos EM . Patterns of musculoskeletal pain in the population: a latent class analysis using a nationally representative interviewer-based survey of 4817 Danes. Eur J Pain. 2013;17(3):452-460. 23042697 10.1002/j.1532-2149.2012.00225.x

[12] Hawkins D Metheny J . Overuse injuries in youth sports: biomechanical considerations. Med Sci Sports Exerc. 2001;33(10):1701-1707. 11581555 10.1097/00005768-200110000-00014

[13] Hirano A Fukubayashi T Ishii T Ochiai N . Magnetic resonance imaging of Osgood-Schlatter disease: the course of the disease. Skeletal Radiol. 2002;31(6):334-342. 12073117 10.1007/s00256-002-0486-z

[14] Huguet A Tougas ME Hayden J McGrath PJ Stinson JN Chambers CT . Systematic review with meta-analysis of childhood and adolescent risk and prognostic factors for musculoskeletal pain. Pain. 2016;157(12):2640-2656. 27525834 10.1097/j.pain.0000000000000685

[15] Kamper SJ Henschke N Hestbaek L Dunn KM Williams CM . Musculoskeletal pain in children and adolescents. Braz J Phys Ther. 2016;20:275-284. 27437719 10.1590/bjpt-rbf.2014.0149 PMC4946844

[16] Kastelein M Luijsterburg PAJ Heintjes EM , et al. The 6-year trajectory of non-traumatic knee symptoms (including patellofemoral pain) in adolescents and young adults in general practice: a study of clinical predictors. Br J Sports Med. 2015;49(6):400-405. 25431450 10.1136/bjsports-2014-093557

[17] King S Chambers CT Huguet A , et al. The epidemiology of chronic pain in children and adolescents revisited: a systematic review. Pain. 2011;152(12):2729-2738. 22078064 10.1016/j.pain.2011.07.016

[18] Kooijman MN Kruithof CJ van Duijn CM , et al. The Generation R Study: design and cohort update 2017. Eur J Epidemiol. 2016;31(12):1243-1264. 28070760 10.1007/s10654-016-0224-9 PMC5233749

[19] Kose O Eraslan A Ergun A Egerci O Ercan E . Prevalence of bipartite patella in Turkish population: analysis of bilateral knee radiographs in 897 subjects. Int J Morphol. 2015;33:1108-1113.

[20] Krul M van der Wouden JC Schellevis FG van Suijlekom-Smit LW Koes BW . Musculoskeletal problems in overweight and obese children. Ann Fam Med. 2009;7(4):352-356. 19597173 10.1370/afm.1005 PMC2713163

[21] Kuehnast M Mahomed N Mistry B . Sinding-Larsen-Johansson syndrome. South Afr J Child Health. 2012;6(3):90-92.

[22] Lasanianos NG Kanakaris NK . Bipartite patella. In: NG Lasanianos, NK Kanakaris, & PV Giannoudis, eds. Trauma and Orthopaedic Classifications: A Comprehensive Overview. Springer London; 2015:453-455.

[23] Lee DW Kim MJ Kim WJ Ha JK Kim JG . Correlation between magnetic resonance imaging characteristics of the patellar tendon and clinical scores in Osgood-Schlatter disease. Knee Surg Relat Res. 2016;28(1):62-67. 26955614 10.5792/ksrr.2016.28.1.62 PMC4779807

[24] Li XH Zhu J Zhang XM , et al. Abdominal MRI at 3.0 T: LAVA-Flex compared with conventional fat suppression T1-weighted images. J Magn Reson Imaging. 2014;40(1):58-66. 24222639 10.1002/jmri.24329

[25] Matic GT Flanigan DC . Return to activity among athletes with a symptomatic bipartite patella: a systematic review. Knee. 2015;22(4):280-285. 26014341 10.1016/j.knee.2015.01.005

[26] Mølgaard C Rathleff MS Simonsen O . Patellofemoral pain syndrome and its association with hip, ankle, and foot function in 16- to 18-year-old high school students: a single-blind case-control study. J Am Podiatr Med Assoc. 2011;101(3):215-222. 21622633 10.7547/1010215

[27] O’Keeffe M Kamper SJ Montgomery L , et al. Defining growing pains: a scoping review. Pediatrics. 2022;150(2):e2021052578. 10.1542/peds.2021-052578 35864176

[28] Paulis WD Silva S Koes BW van Middelkoop M . Overweight and obesity are associated with musculoskeletal complaints as early as childhood: a systematic review. Obes Rev. 2014;15(1):52-67. 23941399 10.1111/obr.12067

[29] Pennock AT Bomar JD Manning JD . The creation and validation of a knee bone age atlas utilizing MRI. J Bone Joint Surg Am. 2018;100(4):e20. doi:10.2106/JBJS.17.00693 29462038

[30] Perquin CW Hazebroek-Kampschreur AAJM Hunfeld JAM , et al. Pain in children and adolescents: a common experience. Pain. 2000;87(1):51-58. 10863045 10.1016/S0304-3959(00)00269-4

[31] Picavet HSJ Berentzen N Scheuer N , et al. Musculoskeletal complaints while growing up from age 11 to age 14: the PIAMA birth cohort study. Pain. 2016;157(12):2826-2833. 27780179 10.1097/j.pain.0000000000000724

[32] Prego-Domínguez J Khazaeipour Z Mallah N Takkouche B . Socioeconomic status and occurrence of chronic pain: a meta-analysis. Rheumatology (Oxford). 2021;60(3):1091-1105. 33276382 10.1093/rheumatology/keaa758

[33] Racunica TL Teichtahl AJ Wang Y , et al. Effect of physical activity on articular knee joint structures in community-based adults. Arthritis Rheum. 2007;57(7):1261-1268. 17907212 10.1002/art.22990

[34] Rathleff MS Rathleff CR Olesen JL Rasmussen S Roos EM . Is knee pain during adolescence a self-limiting condition? Prognosis of patellofemoral pain and other types of knee pain. Am J Sports Med. 2016;44(5):1165-1171. 26792702 10.1177/0363546515622456

[35] Rathleff MS Roos EM Olesen JL Rasmussen S . High prevalence of daily and multi-site pain—a cross-sectional population-based study among 3000 Danish adolescents. BMC Pediatr. 2013;13(1):191. 24252440 10.1186/1471-2431-13-191 PMC3840664

[36] Schellevis F Westert G Bakker D , et al. De tweede Nationale Studie naar ziekten en verrichtingen in de huisartsenpraktijk: aanleiding en methoden. HUWE. 2003;46:956-962. doi:10.1007/BF03083612

[37] Shim KS . Pubertal growth and epiphyseal fusion. Ann Pediatr Endocrinol Metab. 2015;20(1):8-12. 25883921 10.6065/apem.2015.20.1.8 PMC4397276

[38] Thompson LR Boudreau R Hannon MJ , et al. The knee pain map: reliability of a method to identify knee pain location and pattern. Arthritis Rheum. 2009;61(6):725-731. 19479703 10.1002/art.24543 PMC2802101

[39] van den Heuvel MM Oei EHG Renkens JJM Bierma-Zeinstra SMA van Middelkoop M . Structural spinal abnormalities on MRI and associations with weight status in a general pediatric population. Spine J. 2021;21(3):465-476. 33045416 10.1016/j.spinee.2020.10.003

[40] Weiss JE Stinson JN . Pediatric pain syndromes and noninflammatory musculoskeletal pain. Pediatr Clin North Am. 2018;65(4):801-826. 30031499 10.1016/j.pcl.2018.04.004

