# Supplementary material for: Knee Pain, Joint Loading, and Structural Abnormalities on MRI in 13-Year-Old Children in a Population-Based Birth Cohort
Source: Am J Sports Med. 2024 Sep 25;52(12):3046–53. doi: 10.1177/03635465241274792 (PMC11529129; doi:10.1177/03635465241274792)
Supplement: sj-pdf-1-ajs-10.1177_03635465241274792 – Supplemental material for Knee Pain, Joint Loading, and Structural Abnormalities on MRI in 13-Year-Old Children in a Population-Based Birth Cohort [file sj-pdf-1-ajs-10.1177_03635465241274792.pdf]

Knee pain, joint loading and structural abnormalities on MRI in 13-year-old children in a population-based birth cohort

Appendix

Table A1 – Non-response analysis for the study sample

	**Included subjects (n=1849)**	**Excluded subjects (n=4992)**	***P-value***
**Demographics**			
• Sex, boy	891 (48.2)[b]	2548 (51.0)[b]	***0.036***
• Age, years	13.84 [13.61-14.56][b]	13.68 [13.48-14.03][b]	***<0.001***
**Physical factors**			
• BMI, SD score	0.43 {1.19}[b]	0.47 {1.21}[b]	***<0.001***
• Overweight, yes	293 (15.9)	514 (16.7%)	*0.423*
• Height, SD score	0.002 {1.02}	-0.02 {1.02}	*0.393*
• Waist-Hip ratio overweight or obese, yes	506 (28.0)	852 (28.3)	*0.788*
**Physical activity behaviors**			
• Physical activity			
○ minimum 1h/day ≥ 4 days/week, yes	875 (66.1)	1582 (67.6)	*0.347*
• Sports participation, yes	1298 (84.8)	2398 (82.3)	*0.087*
• Active transport, ≥ 1 trip/week, yes	1130 (88.1)	2013 (89.0)	*0.444*
**History of physical activity behaviors**			
• Physical activity at 9, yes	918 (69.3)	1929 (66.4)	*0.068*
• Sports participation at 9, yes	1304 (89.1)	2827 (87.7)	*0.156*

a Values presented as number (%) for categorical factors, or median [interquartile range] or mean {SD} for continuous factors. Bold values represent statistically significant p -values (p<0.05). This table is based on nonimputed data. Missing values were 0 for sex, 1606 (32.2%) for age, 1923 (28.1%) for BMI, overweight and height, 2022 (29.6%) for Waist-Hip ratio, 3177 (46.4%) for physical activity, 2413 (35.3%) for sports participation, 3297 (48,2%) for active transport, 2613 (38.2%) for physical activity at 9 years, 2154 (31.5%) for sports at 9 years.
b Subgroups that significantly differ from each other, based on post hoc analyses.

Figure A1 – Flowchart describing the final study population

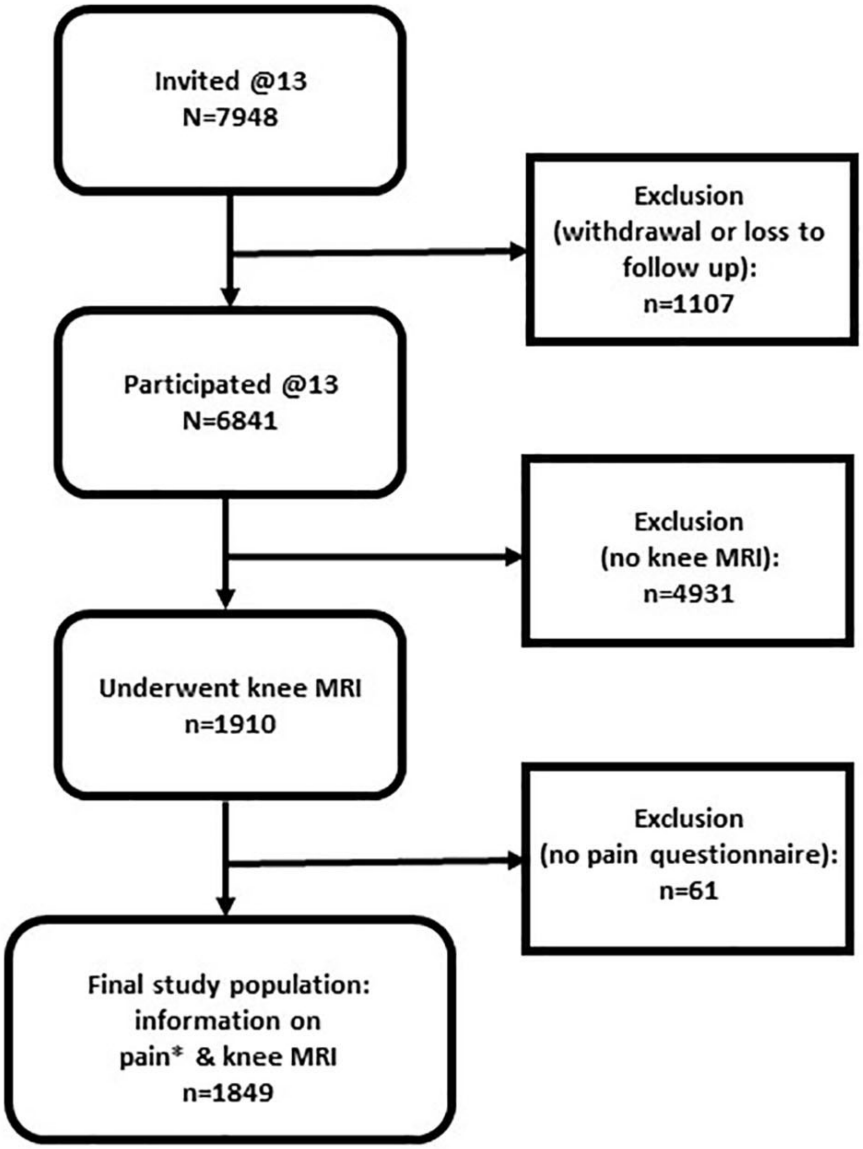

**Additional pain questions on frequency, duration, onset, relation to sports and intensity only available in n=325 children*

Figure A2 – Pain mannequin with 61 possible locations including knee pain

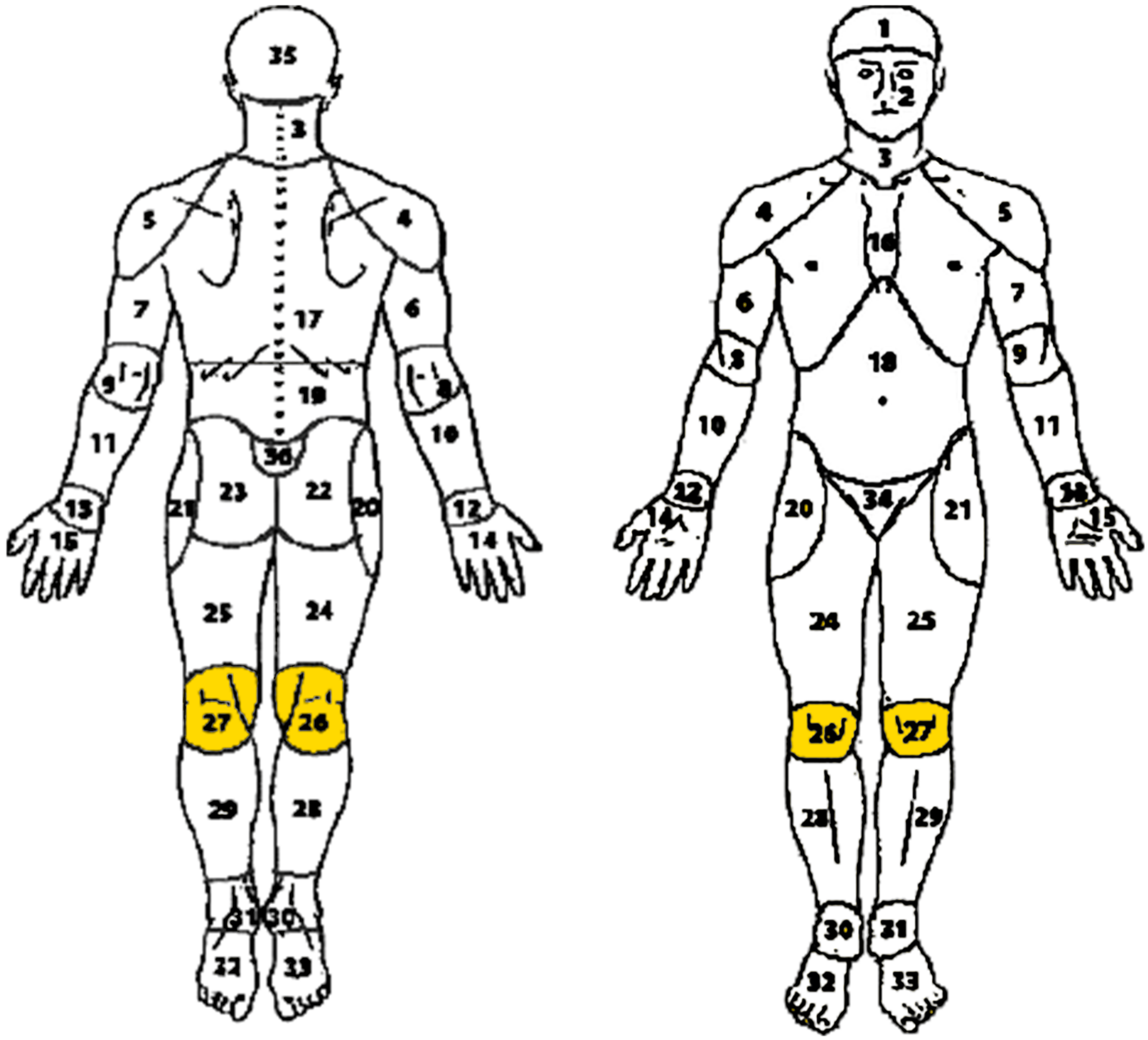

Knee pain (yellow), no knee pain (white)
